# Using Blockchain Technology to Manage Clinical Trials Data: A Proof-of-Concept Study

**DOI:** 10.2196/11949

**Published:** 2018-12-21

**Authors:** David M Maslove, Jacob Klein, Kathryn Brohman, Patrick Martin

**Affiliations:** 1 Department of Critical Care Medicine Queen's University Kingston, ON Canada; 2 School of Computing Queen's University Kingston, ON Canada; 3 Smith School of Business Queen's University Kingston, ON Canada

**Keywords:** blockchain, clinical trial, informatics, data accuracy, data collection

## Abstract

**Background:**

Blockchain technology is emerging as an innovative tool in data and software security.

**Objective:**

This study aims to explore the role of blockchain in supporting clinical trials data management and develop a proof-of-concept implementation of a patient-facing and researcher-facing system.

**Methods:**

Blockchain-based Smart Contracts were built using the Ethereum platform.

**Results:**

We described BlockTrial, a system that uses a Web-based interface to allow users to run trials-related Smart Contracts on an Ethereum network. Functions allow patients to grant researchers access to their data and allow researchers to submit queries for data that are stored off chain. As a type of distributed ledger, the system generates a durable and transparent log of these and other transactions. BlockTrial could be used to increase the trustworthiness of data collected during clinical research with benefits to researchers, regulators, and drug companies alike. In addition, the system could empower patients to become more active and fully informed partners in research.

**Conclusions:**

Blockchain technology presents an opportunity to address some of the common threats to the integrity of data collected in clinical trials and ensure that the analysis of these data comply with prespecified plans. Further technical work is needed to add additional functions. Policies must be developed to determine the optimal models for participation in the system by its various stakeholders.

## Introduction

Clinical trials generate data used in the preparation of peer-reviewed journal papers and applications to regulatory bodies for approval of new treatments. In both cases, the integrity of these data is important to numerous stakeholders, including academic researchers, journal editors and publishers, drug and device companies, government regulators, and most importantly, prospective patients and the general public. For researchers, published papers serve as a key external validator of the rigor of their work and represent important academic achievements. For companies, considerable investment in research and development may be at stake along with the prospect of future earnings, both of which typically count in the billions of dollars. In brief, both publishers and regulators need to trust the data presented to them.

Several threats to the validity of clinical trials data stand to undermine this trust [1]. First, data can be altered or lost, either accidentally or by nefarious intent. Though redundancy exists in many database systems, these are often opaque to outside observers. Second, there is a risk that the published analysis is not a true representation of the analysis that was initially planned [2]. Reasons for variation include failing to report all outcomes measured, selective reporting of positive results (publication bias), stopping data collection after achieving the desired result, and excluding some data after assessing the impact of doing so, among many others. Third, data may be fabricated, manipulated, or duplicated by researchers committing outright fraud. Because of these risks, journals, regulators, and other oversight groups are expected to trust the data generated by clinical trials in the absence of a fully trustworthy relationship with those who generated them.

Blockchain technology has recently emerged as an alternative means for transferring data between participating parties based on a “distributed ledger” model that affords a fully transparent and immutable record of data transactions [3]. A blockchain consists of consecutive chained blocks that are replicated on the nodes of a peer-to-peer network. Blocks consist of records, and each record contains details of a transaction between the users of the system. Blockchain technology was originally designed to serve as the basis for electronic cash systems such as bitcoin [4]; it eliminates the need for trusted third parties in financial transactions by providing a secure and verifiable history for every transaction in the system. Depending on the application, transactions may involve a cryptocurrency, such as bitcoin, or other kinds of assets.

Smart contracts—code that is stored, executed, and verified on a blockchain—are a central component of the next-generation blockchain platforms [5]. Smart contracts can play several roles, including encoding the business logic for an application, ensuring that preconditions for action are met before it is executed, and enforcing permissions for an action. Because smart contracts run on a blockchain, they have unique characteristics compared with other types of software. First, the program itself is recorded on the blockchain, which imparts the blockchain’s characteristic permanence and resistance to censorship. Second, the program can control blockchain assets. Third, the program is executed by the blockchain, meaning it will always execute as written and no one can interfere with its operation.

As blockchain technology matures, applications outside of finance are increasingly being explored, including in the health care sector [6-10]. Blockchain-based models for electronic medical records have been proposed that would empower patients to exercise greater ownership of their medical data and enhance data sharing between platforms [11-13]. Blockchain technology might also prove useful in supporting or even supplanting the traditional data infrastructure used in clinical trials [14-16]. Because the blockchain can be used to establish a permanent record agreed upon by all participating parties, it has the potential to mitigate some of the threats to data validity as outlined above. Immutable clinical trials data recorded using a blockchain may inspire greater confidence in its veracity, resulting in better science, safer medicines, and enhanced public trust in biomedical research.

This study aims to describe BlockTrial, a blockchain system for clinical trials management based on the Ethereum platform. We discuss some design considerations and describe a proof-of-concept system for patient enrollment and data retrieval.

## Methods

**Figure 1:**
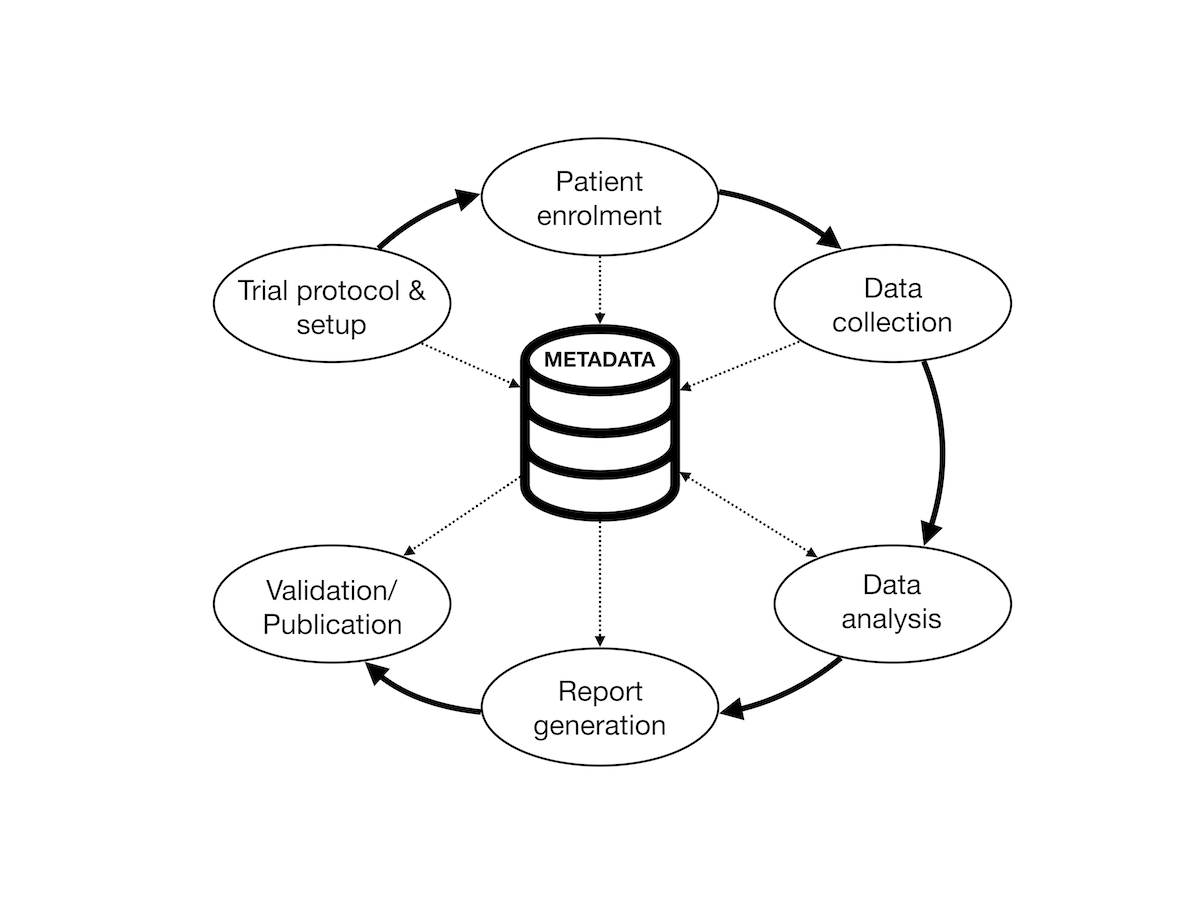
Clinical trials workflow: various phases of the clinical trials process generate and make use of study metadata.

The clinical trials process (Figure 1) consists of trial protocol setup and registration, patient enrollment, data collection, data analysis, report generation, and publication of results [14]. Blockchain solutions may be useful in managing study metadata, including protocol registration, descriptions of prespecified analyses, screening and enrollment logs, and data upload and query logs. The first steps involve the creation of metadata, whereas the later reporting stages refer to these metadata to ensure adherence to protocols and prespecified analyses. Blockchain-based solutions stand to address questions of data integrity and reproducibility at these various stages through the creation of a detailed, time-stamped ledger of data upload and query events.

Our development process addressed 3 main design considerations. The first relates to the type of blockchain that would best suit clinical trials applications. Blockchains can be public, consortium-based, or private. Public blockchains are those that anyone in the world can read and send transactions to, conferring maximal transparency. In this configuration, the nodes extend beyond the research community and could include anyone in the world participating in the public network. Blockchains controlled by a single organization (private) or group of organizations (consortium-based) can have restricted permissions for reading and writing and are therefore only partly decentralized. A consortium-based blockchain might be run by a group of oversight bodies (eg, academic journals and licensing authorities) with nodes distributed across members of the consortium, which in this model, might include investigators active in clinical research; this could, in theory, come at the cost of diminished visibility by outside organizations. Second is the question of who would participate in a blockchain-based clinical trials platform. Several different groups have an important stake in the trials process, including patients, researchers, ethics boards, funders, pharma and device companies, regulatory agencies, publishers, and others. Each of these stakeholders has various interests in the process with differing assets at stake and resources to commit. One advantage of using a blockchain to manage clinical trials is that it stands to directly empower these various actors, who may have substantial investment in the study, be it financial, academic, or personal. The potential to involve patients in the management of their own data is a particularly useful prospect aligned with the ethical principle of autonomy that is central to clinical research. The third is the question of whether the blockchain should store the research data or whether transaction records alone should be stored. For most studies, transaction records would be much smaller in size and could be used to certify the veracity of the data, provide means of determining provenance, and point to clinical and research data stored elsewhere. Moreover, storing transaction records alone on the blockchain allows the platform to exploit existing approaches to managing clinical research data, ensuring compatibility with current clinical trials data management tools such as OpenClinica or REDCap [17]. Although the study data could, in theory, be stored directly on the blockchain, this approach may be impractical because of the limitations of storage space and difficulties in querying the data in such a configuration, especially when considering large datasets such as those generated by genome sequencing.

We developed BlockTrial using a private Ethereum blockchain, allowing us to have full control of the design and development process and leverage the smart contract functionality that is central to our model. Given the abovementioned trade-offs regarding storage space and ease of querying, we developed a model in which the blockchain stores transaction records rather than the clinical trials data itself. Our proof-of-concept platform models the actions of patients who may give or withhold consent to participate in the study, as well as researchers, who collect, store, and analyze the data.

## Results

Ethereum is an open-source programmable blockchain that supports smart contracts and allows developers to create applications at any scale [5]; it includes a peer-to-peer network protocol with each node in the network running the Ethereum Virtual Machine. Operations can be distributed across the network, thereby leveraging the security, accountability, and efficiency of distributed computing. The Ethereum Foundation makes available several Ethereum client implementations, including C++, Python, Go, Java, JavaScript, and Ruby.

**Figure 2:**
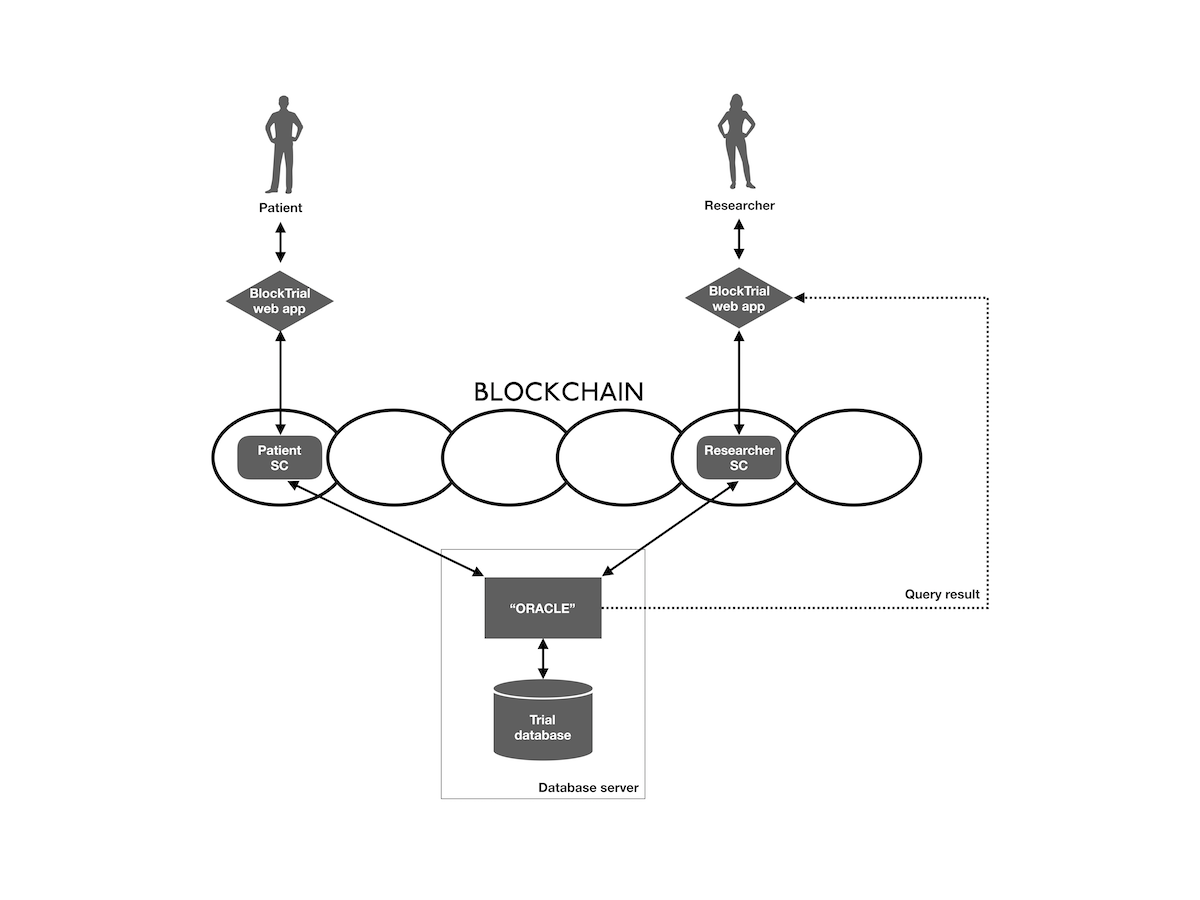
The BlockTrial structure—patients and researchers interact with BlockTrial through the Web-based BlockTrial App. SC: Smart Contract.

Figure 2 shows the structure of BlockTrial. The patient Smart Contract (Patient SC) governs patient enrollment and granting of permissions, whereas the research Smart Contract (Research SC) allows researchers to send and receive queries to the trial database. A blockchain “oracle” is used to interface with “off-chain” resources, including the trial database.

The implementation, which is intended to demonstrate the feasibility of our blockchain-based approach, supports a simplified clinical trial scenario in which patients and researchers interact with one another as well as with the system through a Web app (BlockTrial App). Patient data have been collected and stored in a database that is managed by a *Database Server* outside of the blockchain. Following appropriate discussions with research personnel and upon providing informed consent to participate in a study, patients use the BlockTrial App to register in the study and set permissions on their data for the study. Their registration and permission settings are recorded in the blockchain. Researchers use the BlockTrial App to retrieve study data from the database. Data requests are filtered by patients’ permissions and registered in the blockchain. The requests are in turn retrieved from the blockchain and executed by the database server which sends the results of the requests to the researcher. The data analysis can then be performed outside the system at the researcher’s site.

Smart contracts are placed on the Ethereum blockchain to implement the actions for the patients and researchers. The contracts create transactions to record the actions in the blockchain, thus ensuring the integrity of the study and facilitate requests to the external database server managing the trial database. Furthermore, actions initiated by users through the BlockTrial App are implemented by calling methods made available by smart contracts.

Although smart contracts can run algorithmic calculations, store data, and retrieve data, it is not practical to make arbitrary requests to a service off the blockchain, such as the database server, from a smart contract. Smart contracts interact with the off-chain world through “oracles,” which are agents that watch the blockchain for events and respond to them by invoking a service or performing some action [16]. The database server includes a blockchain oracle that looks for new queries on the blockchain, executes the queries on the database, and publishes the results of the query back to the contract. Textbox 1 shows the methods provided by BlockTrial’s smart contracts.

**Smart contract methods.**

Patient Smart Contract

- addPatient: Registers a patient for the study and sets access permissions
- editPermissions: Changes a patient’s access permissions
- getPeople: Gets the set of patients registered for the study

Researcher Smart Contract

- addQuery: Submits a new query to the blockchain
- addQueryResult: Places a hash of a query result on the blockchain
- getQueries: Retrieves waiting queries from the blockchain
- getUnsolvedCount: Retrieves the number of waiting queries from the blockchain

The patient smart contract (*Patient SC*) maintains an array in the blockchain that holds patients registered for the study and the permissions they have set for their data. Patients register their permission to access data for the study with the *addPatient* method and change their permissions with the *editPermissions* method.

The researcher smart contract (*Researcher SC*) supports researchers issuing queries to the study database and receiving their results. To retrieve study data, a researcher calls the *addQuery* method, which places the SQL text of the query and the address of the researcher issuing the query in a structure on the blockchain maintained by the *Researcher SC*. The researcher then waits for a notification from the database server that the result is available. When the oracle in the database server recognizes that a new query is available in the *Researcher SC*, it uses the *getQueries* method to fetch the query text and researcher address and the *getPeople* method of the *Patient SC* to get the current permissions for all patients registered in the study. The oracle applies the permissions to the query and submits the query to the database. Because query results can be arbitrarily large, it is not practical to put the results on the blockchain. Therefore, we apply a secure hash function, such as SHA-1, to obtain a unique signature that can be used for later validation and append a new transaction to the blockchain containing the query and the result signature. The oracle then makes the result of the query available to the researcher through a separate channel.

## Discussion

### Principal Findings

Clinical trials are one of the cornerstones of biomedical research, providing a mechanism for the objective evaluation of the benefits and risks of new therapies. Trust in clinical trials reporting is synonymous with that in the medical profession itself. Much is at stake, given the potential consequences for patients and their doctors as well as major stakeholders, including researchers, pharmaceutical and device manufacturers, regulators, publishers, and funding agencies. The trustworthiness of clinical trials data is central to modern medical research and practice but for a variety of reasons, it cannot be assumed.

A blockchain platform for clinical trials could be useful in ensuring the trustworthiness of clinical trials data collection and reporting. The need for such a platform reflects the convergence of several trends in clinical research. First, patient autonomy is increasingly recognized as necessary to the conduct of ethical studies and to enhance recruitment into trials [18]. Blockchain technology has already been explored as a means of enhancing agency among patients in the form of electronic medical records that empower patients to share their data with their care providers. A natural extension of this use case is in ongoing consent to participate in clinical trials and to share data with researchers.

Second, regulators such as the Food and Drug Administration often require that data collected in the course of a clinical trial investigating a new drug or device be posted in a registry, such as clinicaltrials.gov, upon study completion [19]. However, adherence to this practice remains poor [20,21]. Blockchain technology could improve adherence to reporting through various forcing functions, particularly at the time that data are queried by investigators for further analysis. Querying the dataset could trigger automatic posting of the data to a registry, perhaps with an embargo period to allow the researchers to complete their initial analysis. In addition, blockchain technology might be useful in ensuring that analysis is done in accordance with the statistical analysis plans that were specified at the time of trial registration. This need reflects a third trend, highlighting the potential negative consequences of multiple post hoc analyses (also referred to as “data dredging”) [22,23].

Finally, blockchain technology suggests a mechanism through which researchers can interact with research oversight bodies, including local research ethics boards. By uploading ethics applications to a blockchain, permanence of the protocol and approval process can be established. Furthermore, a blockchain might facilitate approval by a single research ethics board across multiple sites participating in a multicenter study.

This study described BlockTrial, a proof-of-concept implementation of a blockchain for clinical trials research. BlockTrial operates as a private blockchain, where patients and researchers act as nodes. Patients can assign permissions for which of their data can be viewed, and by whom, whereas researcher requests for data are written into an immutable record. Previous work has explored the use of blockchain technology to enroll patients in clinical trials and record their consent to participate [15,16]. This study extends this model to include research participation in a blockchain with data query and retrieval capabilities. This model empowers patients to take a more active role in their participation in research studies. The Web-based framework stands to improve the efficiency of study enrollment and ensure that consent is as informed as possible. Records of assigning and revoking consent and permission would be visible to all parties and could be used for auditing purposes to ensure proper adherence to trial recruitment guidelines. Immutable records of researcher requests for data and of delivery of said data could be tied to required reporting tasks to ensure that trial registries, such as clinicaltrials.gov, are populated timely with up-to-date and complete data.

This study has several strengths. It uses the existing Ethereum blockchain, which is widely used and which offers several application programming interfaces to facilitate further software development. We model 2 different types of participants, including both patients and researchers as nodes in the network. In addition, we developed a Web-based interface for ease of use. However, this study also has several limitations that must be considered. Chief among these is the cost of participating in an Ethereum-based blockchain; this would require that some participants either purchase ether or mine ether directly (and therefore incur computing and power costs). The optimal distribution of costs remains to be worked out; however, we believe that significant incentives exist for researchers and companies to contribute to these costs, especially if regulations around reporting requirements continue to expand. In addition, our system requires integration with an existing clinical database such as REDCap. Although this does increase the overhead in terms of computing infrastructure and middleware, it is likely to improve the ease and speed of querying data and allows for the use of large data stores, which are unlikely to be easily accommodated on the blockchain itself. The patient-facing functionalities of our model are designed to increase patient autonomy through the ability to assign and revoke permissions for researchers to access data. However, this does introduce the potential that more patients will opt out of a study in progress, potentially introducing considerable selection bias. Finally, the blockchain implementation we describe does not guarantee absolute data integrity; data entry itself remains a point of vulnerability. Although it remains unknown how this model of clinical trial data management will be received by various stakeholders, we believe that substantial benefits may accrue to users of all types and that these would offset the upfront costs of further developing and subscribing to a new system.

Future research and development should address the sociotechnical barriers to implementing blockchain solutions for clinical trials management. Additional participant roles should be modeled, including funders, ethics boards, research institutions, academic journals, trial registries, pharmaceutical corporations, clinical trial organizations, and regulatory agencies. In addition, payment models should be explored, which may include consideration of a specific “BlockTrial coin” cryptocurrency that could be used to run the system and create the necessary incentives to induce the desired participant engagement. Integration with clinical trials registries and journal publishers should be explored to increase data sharing and transparency and facilitate the process of publishing trial results without bias, including selection bias, confirmation bias, and various types of confounding, all of which could be introduced in the course of post hoc analyses. Finally, qualitative research is needed to better describe the attitudes and preferences of patients, researchers, and other stakeholders toward blockchain-enabled clinical trials data infrastructure.

### Conclusions

This study presents a proof-of-concept blockchain-enabled clinical trials data management solution that enables the interaction of patients and researchers engaged in clinical research. BlockTrial affords immediate benefits to patients by empowering them to better control access to their data and to researchers by affording them useful tools to maintain adherence to reporting requirements. Further developed with more stakeholder roles, BlockTrial stands to enhance the integrity of clinical trials data and promote trust throughout the clinical research community and beyond in the output of medical research.

## Abbreviations

SC: Smart Contract
